# Revealing post-transcriptional microRNA–mRNA regulations in Alzheimer’s disease through ensemble graphs

**DOI:** 10.1186/s12864-018-5025-y

**Published:** 2018-09-24

**Authors:** Rubén Armañanzas

**Affiliations:** Department of Bioengineering, Krasnow Institute for Advanced Study, George Mason University, 4400 University Dr, MS2A1, Fairfax, 22030 VA USA

**Keywords:** Post-transcriptional regulation, Ensemble graphs, Bayesian network classifiers, Alzheimer’s disease, Feature stability

## Abstract

**Background:**

*In silico* investigations on the integration of multiple datasets are in need of higher statistical power methods to unveil secondary findings that were hidden from the initial analyses. We present here a novel method for the network analysis of messenger RNA post-translational regulation by microRNA molecules. The method integrates expression data and sequence binding predictions through a set of sound machine learning techniques, forwarding all results to an ensemble graph of regulations.

**Results:**

Bayesian network classifiers are induced based on a pool of ensemble graphs with ascending order of complexity. Individual goodness-of-fit and classification performances are evaluated for each learned model. As a testbed, four Alzheimer’s disease datasets are integrated using the new approach, achieving top values of 0.9794 ± 0.01 for the area under the receiver operating characteristic curve and 0.9439 ± 0.0234 for the prediction accuracy.

**Conclusions:**

Post-transcriptional regulations found by the optimal network classifier concur with previous literature findings. Furthermore, additional network structures suggest previously unreported regulations in the state of the art of Alzheimer’s research. The quantitative performance as well as sound biological findings provide confidence in the ensemble approach and encourage similar integrative analyses for other conditions.

**Electronic supplementary material:**

The online version of this article (10.1186/s12864-018-5025-y) contains supplementary material, which is available to authorized users.

## Background

MicroRNAs (miRNAs) are small noncoding RNA sequences that intervene in the regulation of protein-coding genes after transcription. MiRNAs bind to the 3’UTR region of the target gene to mediate its expression. This regulation varies from full inhibition to degradation of the mRNA product, depending on the sequence complementarity. Currently there exist more than 1,800 sequences identified as miRNAs (miRBase Release 21), and each single miRNA can regulate the production of hundreds of proteins. Therefore, it is no surprise that microRNAs play key roles in the development, differentiation, metabolism, proliferation, and apoptosis of cells. The evidence for their influence in neurological conditions is growing [1, 2]. However, it is still hard to find investigations of differentially expressed genes and microRNAs in human samples of Alzheimer’s disease (AD). Even more scarce is the number of studies on microRNA-mRNA regulations using human biopsies and high-throughput technologies [3] in AD.

A novel *in silico* method for the joint analysis of post-transcriptional microRNA-mRNA regulation is proposed here. The new method is able to combine target sequence binding predictions with differentially expressed profiles. The use of ensemble techniques and stability measures facilitates the integration of multiple datasets of miRNA and/or mRNA expression profiles, introducing an innovative approach to the meta analysis of transcriptional regulations. The use of different microRNA and mRNA datasets contributes statistical power to the data workflow, which ultimately helps unveil new plausible findings. To find the most prominent biological relationships, the core of the analysis is guided by ensembles of Bayesian network classifiers (BNC). BNCs constitute a natural approach to the combination of the data’s structural and functional distributions by fitting the model simultaneously with differentially expressed molecules and predicted genetic interactions.

The new proposal was put to the test in the integration of genomics Alzheimer’s disease datasets. The data curation included the identification of four datasets from common, or adjacent, brain regions to be combined together. This step is crucial in the case of AD research due to the large differences in degeneration shown by brain areas that are resistant versus those submissive to AD spread. Other metadata dimensions were also matched to minimize biases from cognitive state, age, and gender [4]. After the fusion of all datasets into data matrices, we identified those genes with differential expression across phenotypes by comparing control and disease activity profiles. The next step was to feed a group of miRNA target prediction engines with the set of merged miRNAs. Afterwards, a structural score merging all those predictions in a fair way was computed to increase reliability of the detected bindings. A second functional score based on the measurement of conditional mutual information among the triplet, miRNA, gene, and class variable was also computed. This score accounts for the quantitative strength of a miRNA-to-gene interaction over distinct phenotypes. The last step involved the merging of all gathered scores and target interactions through ensemble graphs. These graphs constitute the structural part of Bayesian network classifiers with parameters induced from the expression data. Optimal ensemble classifiers successfully pinpointed previously reported regulations in the AD literature, while also highlighting new ones. In closing, we discuss the grounds of the most significant neurological findings reported by the optimal ensemble.

## Methods

### MicroRNA samples

MicroRNA expression data come from two studies of the temporal lobe both using snap frozen samples [5, 6]. Hébert et al. (2008) [5] performed a differential expression analysis between 5 age-matched controls and sporadic AD samples, respectively (6 female, 4 male). The second work, Wang et al. (2011) [6], analyzed the differential expression of miRNAs between grey and white matter of the temporal gyrus from 10 AD donors (20 samples in total). Due to poor RNA quality, one of the donors was discarded after extraction. Following the authors’ conclusions that patterns of miRNA expression in cortical grey matter may contribute to AD pathogenetically, we retained expression data from the remaining 9 gray matter samples (all female). There were no age outliers among the combined samples, and the final distribution of samples was 5 controls and 14 AD.

### Messenger RNA samples

Two gene expression datasets of mRNA extracted from snap frozen postmortem temporal lobe tissues were combined [7, 8]. Both studies employed the Affymetrix Human U133 Plus 2.0 genechip (54,675 probe sets) platform. Samples in [7] span six brain regions relevant to AD (histopathologically or metabolically). However, we made use only of the medial temporal gyrus samples to maintain neurological coherence in the mixing process.

Subjects were age-matched in both studies independently to avoid bias associated with young subjects. In the combined pool, the lower age limit was 63 years (first quartile minus 1.5 of the interquartile range), whereas the upper limit corresponded to 95 years (third quartile plus 1.5 of the IR). One female control was 102 when she passed away and, although falling out of range, we decided to include it due to the great interest to keep an older subject in the control group. We refer the reader to the original references for extended subjects information and wet-lab procedural details [7, 8]. The final set of mRNA samples included 17 controls and 23 AD cases (15 female, 25 male). Control samples ranked between 0 and 1 in the Braak scale, whereas AD samples were neuropathologically confirmed.

### Combined expression profiling

The set of retained genechips were combined using the *AnyExpress* toolkit [9]. AnyExpress allows the merging of transcript expression profiles coming from a wide range of platforms by mapping them into the associated genomic sequence. A total of 18,216 genetic sequences could be mapped into known gene sequences from the 40 mRNA genechips. The expression levels were normalized by quantile-normalization to remove systematic bias [10, 11]. There exist many well-known techniques to identify differentially expressed genes. Five of them were used for ranking relevant genes across different phenotypes: t-test, permutation, and Wilcoxon hypothesis tests, LIMMA linear model [12], and significance analysis of microarrays (SAM) [13]. A single run of any of those ranking methods could be biased due to the limited set of genechips. Therefore, we integrated a randomized bootstrap resampling method to minimize underpowered results. To compile the final list of relevant genes, we first investigated which of the methods produced more reliable outputs by measuring the stability of the gene rankings when including the bootstrap approach [14]. The empirical cumulative distribution of the number of times a gene had an associated *p*-value lower or equal to 0.05 among all the bootstrap runs was evaluated. Genes in the 95% quantile of this distribution were kept for further analysis.

We found several issues when integrating the microRNA expression profiles. First, data from [6] included multiple lost values and only those miRNA probes with more than 90% of presence were kept. The remaining lost values (20 out of 3,006) were imputed by computing the weighted mean of the three nearest-neighbor probes [15]. Secondly, due to differences between hybridization platforms, the matching of probe identifiers had to be done manually. Hébert et al. (2008) included a total of 328 miRNAs, whereas Wang et al. (2011) included 334 sequences, but only 149 miRNAs could be manually mapped to the same sequence and hence retained for further analyses. Both expression datasets –genes and miRNAs– were log2 transformed and corrected for bias/artifacts using the XPN toolkit [16]. Final merged and parsed datasets are available for download as R-Data files through the additional files section as Additional files 1 and 2.

### Target prediction score for microRNA-gene interactions

Computationally-based algorithms to predict relationships between microRNAs and genes are in constant development. This bioinformatic problem is especially difficult due to the low number of wet-lab validated miRNA-gene associations. Such lack of a priori information leads to many mathematical approaches using different rules of targeting sequence matching between a miRNA and the gene’s promoter region [17]. We chose five well established engines that covered a wide range of computational approaches for target prediction: TargetScan [18], PITA [19], doRiNA (former PicTar) [20], DIANA [21], and miRanda [22]. Target prediction interactions can be translated to an undirected graph where two sets of nodes, microRNA and genes, are connected by edges. Each miRNA in the graph would bind to a subset of genes, and each gene would be conversely related to a subset of miRNAs.

Due to differences in their mathematical and biological grounds, we found a high degree of discrepancy in the predictions retrieved by each engine. Based on this fact, five different graphs connecting our list of miRNAs and genes were computed, and we developed an ensemble miRNA-gene target prediction score to retain only the most reliable interactions.

Each predicted interaction was interpreted as two vertices and an edge in a graph. The selection of the most relevant interactions can be formally modeled as a feature subset selection problem where each edge *e*_*i*,*j*_, mapping the link between miRNA *i* and gene *j*, is seen as a feature out of all possible interactions. As such, it is possible to compare the similarity degree between two miRNA-gene graphs by using a consistency metric that quantifies the degree of (dis)similarity between the two sets of edges. Let *X* be the whole set of edges, with *A* and *B* as two non-empty subsets of it, so that $A,B \subsetneq X$. And let *n*=|*X*| be the total number of edges in *X*. A consistency index *CI*(*A*,*B*) between two subsets *A* and *B* of different cardinalities can be then defined as,

1 $$ CI(A,B) = \frac{rn - k_{M}^{2}}{k_{M}\left(n-k_{M}\right)} \; {,}  $$

where *r*=|*A*∩*B*| is the cardinality of *A*∩*B*. This score takes into account the differences in the number of acquired predictions by selecting the highest cardinality between the two subsets, *k*_*M*_= max{*k*_*A*_,*k*_*B*_}, *k*_*A*_=|*A*|, *k*_*B*_=|*B*|. *CI*(*A*,*B*) varies between −*k*_*M*_ and 1, where values lower than -1 map an almost total dissimilarity and 1 maps a perfect match [14].

Using all possible pairwise comparisons between different graphs, we define the weight set **w** as the set of *m* individual weights *w*_*i*−*j*_ between graphs *i* and *j*. Each element *w*_*i*−*j*_ is computed as the ratio of the consistency score between graphs *i* and *j* and the total sum. The ultimate goal is to produce an ensemble score for all predicted edges where edges frequently included across graphs have a higher score than those seldom reported. The degree of similarity between graphs must also be taken into account, giving priority to edges included in graphs with high consistency across the whole set. Table 1 presents the pseudocode to compute a target prediction score *b* that accounts for all mentioned merging aspects.

**Table 1:** Algorithm for computing the miRNA-gene target prediction score *b*

**Require:G** a set of target prediction graphs, and **w** the associated pairwise consistency weights
$\mathbb {E} \Leftarrow $ Union of all edges in **G**
**repeat**
Extract $e \in \mathbb {E}$
**if** *e* belongs to more than one graph from **Gthen**
$~~~~~~~~~~~~~~~b_{e} \Leftarrow \sum w_{i-j} \; \forall i,j \mid e \in G(i)$ and *e*∈*G*(*j*)
**end if**
$\mathbf {until} \mathbb {E} = \emptyset $

If an edge is included only in one graph, the edge is discarded as spurious by the algorithm in Table 1. Otherwise, the algorithm returns a weight *b*_*e*_ for any given edge *e*. The algorithm assigns higher weights to those edges appearing within graphs whose consistency indexes are also high, i.e., the more similar two predicted graphs are, the more reliable their knowledge is considered. If an edge *e* was included throughout all graphs, its ensemble score *b*_*e*_ will be the maximum (a value of one).

### Mutual information score for microRNA-gene interactions

The information of which profiles come from a control and which from an AD sample, also known as the phenotypic distribution, constitutes a highly valuable metadata to be combined with the expression data of each miRNA and gene. It follows the concept of conditional independence between duplets and triplets of variables which will help integrate these three elements.

In statistics, two continuous variables are independent if the joint density function can be expressed as the product of the marginals. Let *X* and *Y* be two random variables with joint density *f*_*XY*_ and marginals *f*_*X*_ and *f*_*Y*_; *X* and *Y* are independent when *f*_*XY*_(*x*,*y*)=*f*_*X*_(*x*)*f*_*Y*_(*y*) for all values *x* and *y*. As for the expectation, it holds that *E*[*XY*]=*E*[*X*]*E*[*Y*]. It is easy to extrapolate that under the same assumption, *Cov*(*X*,*Y*)=*E*[*XY*]−*E*[*X*]*E*[*Y*]=0. When the independence assumption does not hold, the covariance between *X* and *Y* is not null and hence we expect a degree of correlation. The exact degree and sign of such correlation is measured by the Pearson linear correlation, or *ρ*_*XY*_. The use of correlation between expression profiles of miRNA and gene sets to help interpret target predictions has been widely covered in the literature [23]. However, these works analyze both miRNA and gene expression profiles measured on samples from matching phenotypes.

**Fig. 1:**
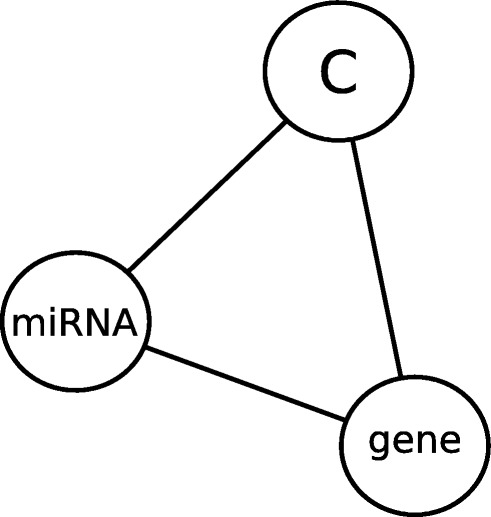
Probabilistic graphical model of a conditional dependency. Conditional dependence structure between a microRNA and a gene, both depending on the class separation

Let *X*, *Y* and *Z* be three random variables. *X* is conditionally independent of *Y* given *Z*, if *f*_*XYZ*_(*x*|*y*,*z*)=*f*_*XYZ*_(*x*|*z*), for all values *x*, *y* and *z*. This definition can be parsed to a probabilistic graphical model through a minimal I-Map of the conditional (in)dependencies. Figure 1 shows the undirected graph which corresponds to such I-Map in the context of miRNA and gene interactions.

Our goal is to test whether the edge between a miRNA *X* and its target gene *Y* is fulfilled by the expression data including the phenotypic distribution of the samples. In order to do so, we used the conditional mutual information *I*(*X*,*Y*|*Z*) between *X* and *Y* given a discrete-valued class *Z*, where

2 $$ I(X,\!Y\!|Z)\! =\! \sum\limits_{z \in \Omega_{Z}}\!\int_{Y}\!\!\int_{X} p(Z)f_{XY}(x,\!y|z) \!\log{\!\frac{f_{XY}(x,y|z)}{f_{X}(x|z) f_{Y}(y|z)}} dxdy.  $$

The conditional mutual information encodes the information that the pair of variables (*X*,*Y*) jointly provide about the value of variable *Z*, where *X*, *Y*, and *Z* are a given miRNA, a gene, and the class variable, respectively. Equation 2 has no closed form in the continuous domain [24]. Instead it is possible to approximate using kernel methods. First, the conditional densities can be rewritten following the conditional rule by expressing them into joint densities and the a priori probabilities of *C* for any possible set of variables **x**:

3 $$ f(\mathbf{x} | c) = \frac{f(\mathbf{x}, c)}{f(c)} \; = \frac{f(\mathbf{x}, c)}{p(c)} \; {.}   $$

The joint and marginal continuous densities can then be estimated from data using uni- and bi-variate Gaussian kernel density estimations as follows. Given a dataset $\mathcal {D} = \left \{\mathbf {x}^{(1)}, \dots, \mathbf {x}^{(N)}\right \}$ with *N* instances of *n*-dimensional vectors $\mathbf {x}^{(j)} = \left (x_{1}^{(j)}, \dots, x_{n}^{(j)}\right)$, the *n*-dimensional kernel density estimator is defined as

4 $$ f(\mathbf{x}; \mathbf{H}) = \frac{1}{N}\sum\limits_{j=1}^{N}{K_{\mathbf{H}}\left(\mathbf{x} - \mathbf{x}^{(j)}\right)} \; {,}   $$

where **H** is a *n*×*n* bandwidth matrix, *K*_**H**_(**x**)=|**H**|^−1/2^*K*(**H**^−1/2^**x**) and *K*(·) is the kernel function. We here used the Gaussian kernel and the kernel’s bandwidth **H** was estimated following the normal reference rule [25].

The microRNA-gene mutual information score *d*_*e*_ is therefore defined as the conditional mutual information *I*(*X*,*Y*|*C*) for the edge *e* linking miRNA *X* and gene *Y*, given the distribution of the class variable *C*.

### MicroRNA–mRNA regulatory network

All edges identified by the target prediction algorithm have two scores: a target prediction score *b*, also referred to as *structural score*, and a *functional score* or mutual information score *d*. We here detail the process to induce and assess the performance of Bayesian network classifiers based on both scores. It follows an introduction on probabilistic graphical models (PGM), the Bayesian network classifiers (BNC) induced to map the micro-mRNA interactions, and the metrics used to choose the best network out of all the induced graphs.

#### Probabilistic graphical models

Probabilistic graphical models (PGM) represent multivariate joint probability distributions via a product of terms, each of which involves only a few variables. The structure of this product is represented by a graph that relates variables that appear in a common term. This graph specifies the product form of the distribution and also provides tools for reasoning about the properties entailed by the product. PGMs based on directed acyclic graphs (DAG) make use of the concept of conditional independence to obtain the joint probability distribution. When a graph fulfills the constraints to be considered a DAG [26], the structure of the associated PGM can be assumed to follow an ancestral ordering where each node *X*_*i*_ takes the *i*-th position in that ordering. Thus, for every ancestral node *X*_*j*_ of *X*_*i*_, we can state that *j*<*i*.

Formally, let **G**=(**X**,**L**) be the DAG of a PGM that follows an ancestral ordering, the set of parents of a node *X*_*i*_, **p***a*_*i*_, *D*-separates *X*_*i*_ from any previous node in the ancestral ordering. Consequently, *X*_*i*_ is conditionally independent of any *X*_*j*_, with *j*<*i*, given the value of its parents. Combining this property with the chain rule, it is possible to induce the joint probability distribution encoded by **G** as $ f(\mathbf {x}) = \prod _{i=1}^{n} f(x_{i}|\mathbf {pa_{i}},\mathbf {\theta }) \text {,}$ where **θ**∈***Θ*** is the set of parameters associated with each node.

#### Bayesian network classifiers

A Bayesian network is a PGM fully described by a directed acyclic graph **G** and the set of parameters **θ** associated with the probability distributions of each variable *X*_*i*_ in **G**. The use of Bayesian network structures for classification tasks give rise to what is broadly known as Bayesian network classifiers. The majority of BNCs assume that the class variable is parent to all predictive variables, also known as features. BNCs are generative classifiers that encode the joint probability distribution of the data through the graphical dependences of the Bayesian network. In classical BNCs, the output classes are exclusive, i.e., the class variable *C* can only take one of its *k* possible values {*c*_1_,…,*c*_*k*_}.

To compute the probability distribution of the predictive variables, it is common practice the use of normal densities [27, 28]. Most BNCs with continuous predictive variables conform with the class of conditional linear Gaussian networks [29] where the conditional probability density *f* (*x*_*i*_|**p***a*_*i*_) is modeled using a conditional linear Gaussian density for each variable *X*_*i*_. The set of values in **pa**_*i*_ depends on the continuous densities of the parent variables **y**_*i*_, as well as on the discrete distribution of the supervised class. This kind of classification models are called *conditional linear Gaussian classifiers* [28] in which two kinds of conditional dependences are allowed: arcs between pairs of continuous features, and arcs between the discrete class and the features. Let *p*(*c*),*c*∈*Ω*_*C*_ with ${\sum \nolimits }_{c=1}^{K}{p(c)}=1$ be the categorical distribution of the class variable *C*, and let each feature *X*_*i*_ have parents **p***a*_*i*_={**Y**_*i*_,*C*} with **Y**_*i*_⊆**X**∖{*X*_*i*_}. It is then possible to define the conditional linear Gaussian density functions *X*_*i*_|**y**_*i*_,*c* as

5 $$ f_{X_{i}|\mathbf{y}_{i},c}(x_{i}) = \mathcal{N}\left(\beta_{0X_{i}|\mathbf{Y}_{i},c} + \boldsymbol\beta_{X_{i}|\mathbf{Y}_{i},c}^{T} \, \mathbf{y}_{i}, \sigma_{X_{i}|\mathbf{Y}_{i},c}^{2}\right),  $$

where

$$\begin{aligned} \beta_{0X_{i}|\mathbf{Y}_{i},c} &= \mu_{X_{i}|c} - \boldsymbol\Sigma_{X_{i}\mathbf{Y}_{i}|c}\boldsymbol\Sigma_{\mathbf{Y}_{i}|c}^{-1}\boldsymbol\mu_{\mathbf{Y}_{i}|c} \\ \boldsymbol\beta_{X_{i}|\mathbf{Y}_{i},c} &= \boldsymbol\Sigma_{\mathbf{Y}_{i}|c}^{-1} \boldsymbol\Sigma_{\mathbf{Y}_{i}X_{i}|c} \\ \sigma_{X_{i}|\mathbf{Y}_{i},c}^{2} &= \Sigma_{X_{i}|c} - \boldsymbol\Sigma_{X_{i}\mathbf{Y}_{i}|c}\boldsymbol\Sigma_{\mathbf{Y}_{i}|c}^{-1}\boldsymbol\Sigma_{\mathbf{Y}_{i}X_{i}|c} \end{aligned} $$

The joint density function encoded by the graphical structure of **G** and the set of **X** continuous features is given by the following finite mixture model

6 $$ f(\mathbf{x}) = \sum\limits_{c=1}^{k} p(c) \prod\limits_{i=1}^{n}{f_{X_{i}|\mathbf{y}_{i}, c}\left(x_{i} \,;\, \beta_{0X_{i}|\mathbf{Y}_{i},c}, \boldsymbol\beta_{X_{i}|\mathbf{Y}_{i},c}, \sigma_{X_{i}|\mathbf{Y}_{i},c}^{2} \right)} \; {.}  $$

When classifying a new instance **x**∈*Ω*_**X**_, a BNC yields a posterior probability *p*(*c*|**x**) for each class label *c*∈*Ω*_*C*_. Then, the maximum a posteriori decision rule is used so that **x** is assigned to the class *c*^∗^ with maximum posterior probability. For conditional linear Gaussian networks it is computed as

7 $$ c^{*} = \arg \max_{c \in \Omega_{C}}{p(c|\mathbf{x})} = \arg \max_{c \in \Omega_{C}}{p(c)\prod\limits_{i=1}^{n}{ f_{X_{i}|\mathbf{y}_{i}, c} }} \; {,}   $$

where *p*(*c*) is the prior probability of class value *c*∈*Ω*_*C*_ and $f_{X_{i}|\mathbf {y}_{i}, c}$ are the conditional density functions computed from Equation 5.

#### Network validation

Provided with sufficient data to be statistically representative, theory can define the most powerful explanatory model based on the goodness-of-fit of a model to a given dataset. However, due to the usual shortage of data, the choice of the best model through the maximum likelihood score usually carries the addition of artifacts, either on variables and/or relationships. It is common practice to balance the model’s fit to the data with the incorporation of a penalization term. We here propose the use of the Bayesian information criterion (BIC score) that accounts for the trade-off between model complexity and goodness-of-fit by including a penalization term based on the number of variables and parameters to be estimated. Formally, let $\mathcal {D}$ be the dataset, $\mathcal {S}$ a given model formed by *N* variables describing $\mathcal {D}$, and *θ* the set of specific parameters, the BIC score is defined as

8 $$ BIC = \mathcal{L} (\mathcal{D} \mid \mathcal{S},\theta) - pen (\mathcal{N}) dim(\mathcal{S}) \; {,}  $$

where $\mathcal {L} (\mathcal {D} \mid \mathcal {S},\theta)$ is the log-likelihood of $\mathcal {S}$ and *θ* to $\mathcal {D}$, $pen (\mathcal {N})$ = *log*(*N*)/2, and, $dim(\mathcal {S})$ is the number of parameters to estimate.

Classification error and/or accuracy are the most frequently used performance measures for illustrating the goodness of a classification model. The accuracy of a given classifier, *Acc*_*γ*_, is the probability of correctly classifying a new instance **x**: $ Acc_{\gamma } = \sum _{\mathbf {x}}p(\gamma (\mathbf {x})= c)p(\mathbf {x})$. The dual nature of BNCs as Bayesian networks and pattern recognition models allows the estimation of both figures of merit, accuracy and BIC score. Accuracy is a fair measure to evaluate goodness-of-fit when the error cost is equally distributed for all classes and the dataset is balanced. To avoid those biases, we also computed a more generalized metric for binary classification, the area under the ROC curve or AUC [30]. AUC shows the ability of a model to give good relative scores to the observations and it is an equivalent to the Wilcoxon test of ranks for classification. It is possible to compute the AUC value of a model by using an average of a number of trapezoidal approximations such that $AUC = \frac {1}{2} {\sum \nolimits }_{k=1}^{n} \left (X_{k} + X_{k-1}\right)\left (Y_{k} - Y_{k-1}\right)$, where (*X*_*i*_,*Y*_*i*_) = (*FPR*_*i*_,*TPR*_*i*_) are the false positive and true positive rates for the *i*-th classification instance, respectively.

## Results

### Differentially expressed mRNA

After merging the expression profiles, the mRNA dataset included 40 cases with 18,216 transcripts mapped into known gene sequences. Five well-known differential expression algorithms were then considered to filter irrelevant genes: t-test, LIMMA, SAM, permutation test, and Wilcoxon test. To establish which would perform best for our dataset, we computed stability scores over a 1,000 resampled bootstrap runs. Table 2 shows the stability scores for each individual method using weighted Spearman correlation as the similarity index between rankings computed over the bootstrapped datasets. SAM reached the highest median and mean stability scores, showing similar dispersion measurements to the other methods. These results are in accordance with previous findings on the good stability properties of the SAM statistic for differential expression analyses [31]. SAM tends not to select genes with small fold changes and/or high variances among the replicates and it does not rely on a priori assumptions about the data probability distribution. Therefore, SAM was chosen as the method to filter out irrelevant genes. SAM generated an associated *p*-value for each gene and ranking out of the 1,000 rankings produced in the bootstrap resampling. We then computed the empirical cumulative distribution (CDF) of the number of times a gene had an associated *p*-value lower than or equal to 0.05. The empirical CDF was composed by 897 unique values with the following value summary: Minimum value 16, maximum value 984, first quartile 240, third quartile 689, median value 464, and mean value 467. We set the 95% quantile (a value of 645.25) as the cutoff point [32], retaining a total of 911 genes.

**Table 2:** Stability scores of weighted Spearman correlation of a pool of 1000 differentially expressed rankings computed from class-balanced 1000 bootstrapped mRNA databases

	Min.	1 ^*st*^ qu.	Median	Mean	3 ^*rd*^ qu.	Max.
T-test	-0.8050	-0.2540	0.0683	0.0044	0.2860	0.6570
LIMMA	-0.7960	-0.2160	0.1030	0.0346	0.3140	0.6690
SAM	-0.7910	-0.1740	**0.1400**	**0.0683**	**0.3470**	**0.6790**
Permutation	-0.7990	-0.2700	0.0508	-0.0117	0.2610	0.6340
Wilcoxon	**-0.8380**	**-0.2740**	0.0077	-0.0360	0.2350	0.6660

Values of -1 or 1 correspond to the highest concordance and 0 to the lowest. Highlighted in bold are the highest absolute values

### Target prediction and mutual information scores

Table 3 presents the final summary figures for the five binding prediction algorithms, namely TargetScan, doRiNA, DIANA, miRanda, and PITA. All predictions were combined into the structural score *b* following the algorithm in Table 1. The process returned a total of 32,004 unique edges with structural weights ranging from a minimum of 0.0551 to a maximum value of 1. For clarity, the prefix ’*hsa-*’ was removed from all microRNA identifiers. Details on the prediction process follow.

**Table 3:** Statistics of microRNA-gene binding site predictions

	Predictions	Unique	Gene	MicroRNA
TargetScan	30,703	22,173	GPR26 (101)	miR-520d-5p (403)
doRiNA	–	1,395	LCOR (16)	miR-137 (73)
DIANA	47,114	27,967	PSD3 (97)	miR-495 (438)
miRanda	41,155	24,437	UHRF2 (89)	miR-186 (419)
PITA	86,996	41,600	LONRF2 (130)	miR-186 (517)

Column *Predictions* shows the total number of predicted interactions, with repetitions removed in column *Unique*. *Gene* and *MicroRNA* columns include which gene and microRNA received the highest number of interactions

The 3’ UTR sequences of 30,888 human genes reported by the TargetScan database (release 6.2) and the sequence of 1,539 human microRNA were initially retrieved. We filtered out those microRNA and gene sequences which unmatched our previous filtering stages. As a result, there were 863 3’ UTR gene sequences and 143 microRNAs. TargetScan detected 30,703 prediction matches among the subset of miRNAs and genes with different degrees of sequence binding, with a total number of 22,173 unique relationships. The next targeting engine was doRiNA, formerly known as PicTar. doRiNA directly accepts a list of mRNA target HGNC identifiers and predicts the binding of a predefined list of human microRNAs by means of the UCSC database. From our list of 911 differentially expressed mRNA, doRiNA correctly matched 761 of them. doRiNA found a total of 135,171 interactions, however, after filtering to unique doRiNA’s microRNA and mRNA identifiers, the set was reduced to 1,395 individual predictions. The set of DIANA v4.0 precomputed target predictions included 3,541,029 possible bindings among microRNAs and mRNA sequences in homo sapiens. From them, a total of 47,114 jointly bound our subsets of miRNA and genes. By removing redundancies, the final graph of dependencies for DIANA contained 27,967 edges.

The fourth target prediction used was miRanda. Following the authors suggestions [33], we collected homo sapiens predictions with mirSVR score ≤ -0.1 or those with either a 6-mer or better seed site, for both conserved and non-conserved miRNAs. We got a set of 628,480 bindings compatible with our miRNA subset out of 4,417,886 total predicted bindings. From them, only 41,155 predictions were based on our list of miRNAs and genes. After the final filtering, the prediction graph included 24,437 edges. The last prediction engine considered was PITA v6. We downloaded the PITA catalog of predicted microRNA targets provided from the supplementary material in [19]. There were multiple predictions for both human miRNAs and genes up to an overwhelming 7,513,144 hits. From them, 1,645,183 includes predictions of our miRNA list, and a total of 86,966 hits jointly matches our lists of miRNAs and genes with a final 41,600 unique relationships.

The functional score *d* based on mutual information approximation was computed for all possible edges between the identified 911 differentially expressed genes and the 149 available miRNAs profiles. The top ten edges with highest structural and functional scores are presented in Table 4. The diversity between rankings in Table 4 highlights how dependent the prediction is whether expression or structural information is used in the prediction process.

**Table 4:** Top ten mRNA-gene edges ranked based on structural and functional scores

Structural			Functional		
MicroRNA	Gene	*b*	MicroRNA	Gene	*d*
miR-184	PPP1CC	1.0000	miR-106a	KIF1B	0.0206
miR-215	PABPC4	1.0000	miR-106a	ZCCHC2	0.0203
miR-504	GRM3	1.0000	miR-106a	EFHA2	0.0201
miR-142-3p	GNB2	1.0000	miR-106a	EPB41L1	0.0196
miR-142-3p	PSRC1	1.0000	miR-106a	THRB	0.0193
miR-328	ZNF423	1.0000	miR-106a	WASL	0.0192
let-7c	ABCC10	1.0000	miR-106a	ACPL2	0.0191
miR-142-3p	XPO1	1.0000	miR-106a	N4BP1	0.0191
miR-504	CEP170	1.0000	miR-106a	KLHDC5	0.0190
let-7c	CYP46A1	1.0000	miR-106a	CAP2	0.0189

### Optimal post-transcriptional regulatory network

The structure of the ensemble graph was defined following the ranking of edges filtered by their associated structural and functional scores. All edges were sorted using both scores, (*b*_*e*_,*d*_*e*_) for edge *e*, in a multiobjective way by using a Pareto optimality sorting. In order to build a directed acyclic graph for the network classifier, the following iterative procedure was followed: i) in an empty graph add all the nodes connected by the first *t* number of edges in the list; ii) transform each undirected edge into a directed one by pointing out from the miRNA to the gene nodes; iii) include the class node *C* as parent of all nodes in the graph. This procedure assured the acyclic property of the DAGs so the resulting graph mapped a BNC structure. The parameters of the BNC could then be induced from data following the conditional linear Gaussian classifier model.

**Fig. 2:**
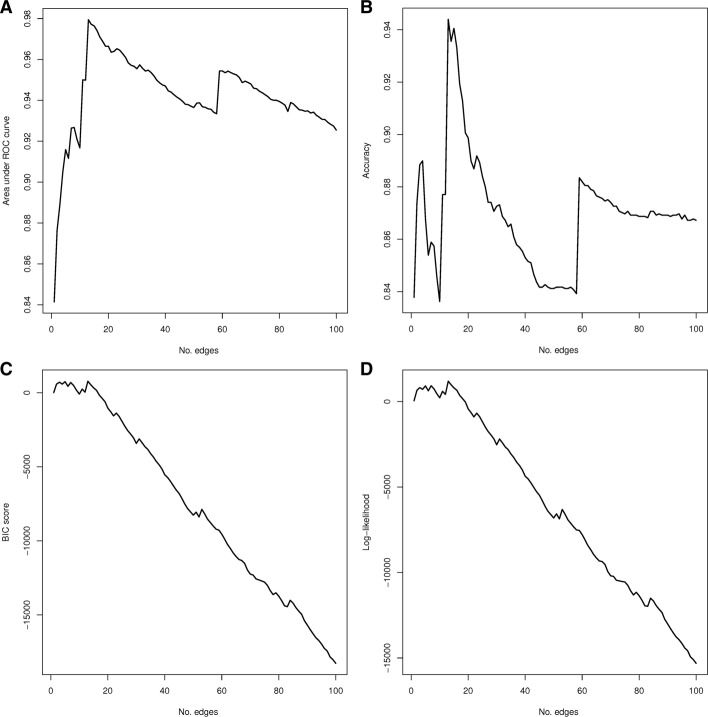
Performance estimation in classification. Average validation metrics for all ensemble Bayesian network classifiers up to 100 edges. From left to right and top to bottom: AUC, Accuracy, BIC, and Log-likelihood **a** Area under ROC curve **b** Accuracy **c** BIC score **d** Log-likelihood

**Fig. 3:**
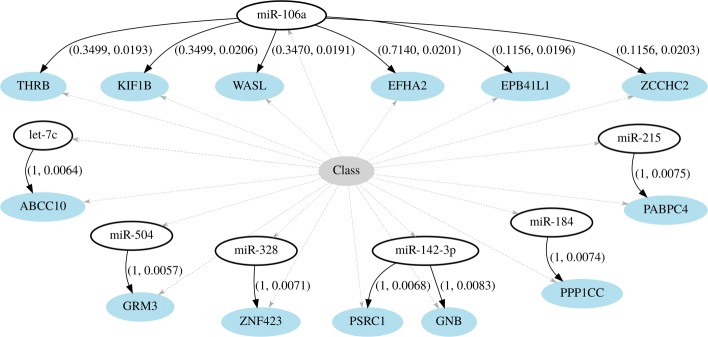
Optimal ensemble structure of post-transcriptional microRNA–mRNA regulations. Optimal ensemble classification structure comprised by 13 edges, connecting 7 miRNAs and 13 genes. Labels over edges include the pair of structural and functional weights (*b*,*d*) for each dependence

The remaining question was which of all possible classifiers performed best when varying the threshold *t*. Each BNC was numerically assessed through a five-times five-fold cross-validation. This validation scheme was proven to be well-suited for the microarray context [34], guaranteeing a fair and not overfitted performance assessment. The validation process started from the simplest BNC formed by one edge (*t*=1) and three nodes (one miRNA, one gene, and the class variable) and grew iteratively. To evaluate the goodness-of-fit of each BNC, four different criteria were computed: AUC score, classification accuracy, BIC score, and log-likelihood of the data. Figure 2 shows average results for each criteria when evaluating BNCs up to 100 edges. The four criteria reached the same maximum peak when the BNC included 13 edges with 0.9794 ± 0.010 for AUC, 0.9439% ± 0.0234 in accuracy, a BIC score of 771.993 ± 63.194, and 1186.471 ± 63.194 for log-likelihood. The network structure corresponding to this optimal BNC is shown in Fig. 3, including individual scores (*b*_*e*_,*d*_*e*_) for each edge *e*. More complex models (*t*>13) showed a significant decay in performance no matter how many new edges were added or which criterion was considered.

## Discussion

Control of transcription by microRNA molecules is currently known to be a key process in the development of sporadic Alzheimer’s disease. The first target in this search was to elucidate whether miRNAs could participate in the regulation of the amyloid precursor protein (APP) and/or its precursor gene. Amyloid-beta peptide is generated by the amyloid precursor protein through the *amyloidogenic* pathway with the help of beta and gamma secretases. In Alzheimer’s, the excessive accumulation of amyloid-beta peptide in extracellular spaces forms what is known as the beta-amyloid plaques, one of the hallmarks of Alzheimer’s disease. Hébert et al. (2008) showed that miRNAs belonging to the miR-17 family (i.e., miR-17, miR-20a, miR-106a and miR-106b) could regulate APP expression in vitro and at the endogenous level in neuronal cell lines [35]. Complementary works corroborated those results, one showing how the miR-17 family directly suppresses APP in vitro, and another reporting a statistically significant decrease in miR-106b expression in sporadic AD patients [1, 36]. These findings are in accordance with the finding by Patel et al. (2008) that over-expression of miR-106a in human cell lines is known to negatively regulate reporter gene expression of the amyloid precursor protein, resulting in translational repression and reduction of APP protein levels [37]. Accordingly, the miR-106a molecule is ranked highly relevant by the functional score (see Table 4), and has a prominent place in the optimal BNC structure of Fig. 3, corroborating its major importance for AD.

Three of the genes under the direct influence of miR-106a in our network are of key interest: the kinesin family member 1B (KIF1B), the thyroid hormone receptor beta (THRB), and the Wiskott-Aldrich syndrome-like (WASL). WASL encodes a member of the Wiskott-Aldrich syndrome (WAS) protein family. It interacts with several proteins involved in cytoskeletal organization and is highly expressed in neural tissues. The relationship between WASL and AD is well-known based on the Tg2576 mouse model of AD. Brains of Tg2576 mice overexpress a mutant form of the APP gene, resulting in elevated levels of APP and ultimately in amyloid plaques. The WASL gene is among a number of genes being down-regulated by the mutations in Tg2576 models, and its boosting is known to restart neuronal regeneration [38]. A similar process was reported in the AD literature for other members of the WASL family [39]. THRB serves several key neurodevelopmental roles, with special stress on the mediation of biological activities of the thyroid hormone. Pair-wise analyses have shown significant correlations between THRB and neuroserpin, a serine proteinase inhibitor that plays a pivotal role in the synaptogenesis of learning, memory, and behavior. The up-regulation of neuroserpin in Alzheimer’s disease brains may result from an activation of the thyroid hormone response [40], which is in accordance with our findings. Lastly in this group, KIF1B encodes a brain motor protein that transports mitochondria and synaptic vesicle precursors and it is linked with AD through the subcellular trafficking of APP [41].

Our results also corroborated the importance of miR-504. This microRNA is a tumor-suppressive molecule produced by the chromosome X, and is strongly linked to various cancers, especially glioma brain tumors. miR-504 shows dysregulation in bipolar disorder and other neuropsychiatric disorders [42] where it alters the density of dopamine receptors in the brain [43]. Its companion in the network, the gene GRM3, also produces metabotropic receptors, but of glutamate neurotransmitters. Glutamatergic neurotransmission is involved in most aspects of normal brain function and can be perturbed in many neuropathologic conditions. GRM3 is well-known to be implicated in the pathophysiology of schizophrenia, for instance. However, to the best of our knowledge, no other study has linked miR-504 and GRM3 with the neuropathology of AD. Dopaminergic disturbances in the brain can lead to glutamatergic receptor changes [44] and vice versa [45], corroborating the regulatory dependence identified here.

Another notable molecule in the network of Fig. 3 is miR-142-3p, involved in the regulation of two genes: proline and serine rich coiled-coil 1 (PSRC1), and G protein subunit beta 2 (GNB2). miR-142-3p was recently flagged as highly relevant for the prediction of AD using plasma samples [2]. It was included within a seven microRNA biomarker panel that distinguished AD samples from control with 95% accuracy and an AUC of 0.953. miR-142-3p was the best stand-alone microRNA in terms of specificity (100%) and sensitivity (65%) as compared to the rest of the panel. For its part, PSRC1 encodes a proline-rich protein that plays an important role in mitosis and has been reported as brain specific with an average of 9.007 reads per kilobase of transcript per million (RPKM) [46]. The second gene, GNB2, is a protein coding gene whose transcript is involved in various transmembrane signaling systems. GNB2 has been found differentially expressed in the anterior cingulate cortex from patients with schizophrenia [47]. As previously discussed with GRM3, this link with schizophrenia comes through the dopamine receptor mediated signaling pathway, which has a total of 59 genes. Remarkably, the network of Fig. 3 includes three out of these 59, namely PPP1CC, EPB41L1, and GNB2.

Higher animals have multiple isoforms of the let-7 family of miRNAs, including let-7c. The members of this family are categorized by a highly conserved consensus sequence whose function is to negatively regulate oncogenes by controlling cell growth genes [48]. The set of microRNAs in the let-7 family receives the pseudo-name of *anti-oncomirs* or *post-transcriptional-gatekeepers* due to their function, and they constitute the first anti-oncomir family ever reported. An example of this regulation is how let-7a and let-7e modulate the gene ABCC10 in some cancers like hepatocellular carcinomas [49]. This particular relation could also explain the link between let-7c and ABCC10 in Fig. 3. In the context of AD, let-7c plays a key role during major periods of neurogenesis in the cortex of mouse embryos and in the postnatal cerebellum [50]. Its influence into AD pathogenesis has been largely proven by analysis of cerebrospinal fluid [51], blood mononuclear cells [52], and other tissues [53].

## Conclusions

The combination of multiple datasets to gain higher statistical power is unfeasible in most cases due to sample and technological variability, incompatible metadata dimensions, and distinct quantitative features. Here we propose a new method to tackle meta-analyses of microRNA-mediated regulation of gene expression during post-transcriptional interactions. Our ensemble pipeline combines structural and functional results in an ensemble graph that ultimately helps overcome the disparity between top-ranked dependences reported by various target prediction engines. It is uncommon to combine results coming from different microRNA target prediction engines, and when done the output is usually a single union or intersection of all those predictions. These simple blends impose higher relevance to engines retrieving a large number of predictions, blurring smaller prediction subsets.

In contrast, more sophisticated ensemble methods are known to produce fairer combinations, and hence enhance the final graph of regulations [54]. Moreover, the evaluation of classification performance and goodness-of-fit scores over increasingly more complex networks maximizes the odds of including relevant findings within optimal models. Our method was put to the test in combining four Alzheimer’s disease genomics datasets. The results showed excellent quantitative performance while matching findings reported in the state-of-the-art biology of AD. The new method also identified new regulations that, to the best of our knowledge, have not been discussed in the current literature. The ensemble of structural and functional findings allows similar analyses in biomedical scenarios beyond the application presented here. Opportunities to combine multiple phenotypes and regulations in comparative medicine are now at hand.

## Additional file

Additional file 1 MicroRNA merged and filtered data R-Data file with the datasets from [5] and [6] merged and filtered following the data processing presented in the “Methods” section. (RDATA 22 KB)

Additional file 2 mRNA merged and filtered data R-Data file with the datasets from [7] and [8] merged and filtered following the data processing presented in the “Methods” section. (RDATA 322 KB)

## Abbreviations

ABCC10: ATP binding cassette subfamily C member 10
AD: Alzheimer’s disease
APP: amyloid precursor protein
AUC: area under the ROC curve
BIC: Bayesian information score
BNC: Bayesian network classifier
CDF: cumulative distribution function
CI: consistency index
DAG: directed acyclic graph
EPB41L1: erythrocyte membrane protein band 4.1 like 1
FPR: false positive rate
GNB2: G protein subunit beta 2
GRM3: glutamate metabotropic receptor 3
HGNC: HUGO gene nomenclature committee
IR: interquartile range
KIF1B: kinesin family member 1B
miRNA: microRNA
mRNA: messenger RNA
PGM: probabilistic graphical models
PPP1CC: protein phosphatase 1 catalytic subunit gamma
PSRC1: proline and serine rich coiled-coil 1
RNA: ribonucleic acid
ROC: receiver operating characteristic
RPKM: reads per kilobase of transcript per million
THRB: thyroid hormone receptor beta
TPR: true positive rate
UCSC: University of California, Santa Cruz
UTR: untranslated region
WASL: Wiskott-Aldrich syndrome-like
